# Neural Differentiation of Mouse Bone Marrow-Derived Mesenchymal Stem Cells Treated with Sex Steroid Hormones and Basic Fibroblast Growth Factor

**DOI:** 10.22074/cellj.2015.509

**Published:** 2015-04-08

**Authors:** Kazem Parivar, Javad Baharara, Azar Sheikholeslami

**Affiliations:** 1 Department of Biology, Sciences and Research Branch, Islamic Azad University, Tehran, Iran; 2 Department of Biology, Faculty of Sciences, Mashhad Branch, Islamic Azad University, Mashhad, Iran; 3 Department of Zoology, Faculty of Biological Sciences, Kharazmi University, Tehran, Iran

**Keywords:** Sex Steroid Hormones, bFGF, Neural Differentiation, Mesenchymal Stem Cells

## Abstract

**Objective:**

There are several factors, like environmental agents, neurotrophic factors, serotonin and some hormones such as estrogen, affecting neurogenesis and neural differentiation. Regarding to importance of proliferation and regeneration in central nervous system, and a progressive increase in neurodegenerative diseases, cell therapy is an attractive approach in neuroscience. The aim of the present study was to investigate the effects of sex steroid hormones and basic fibroblast growth factor (bFGF) on neuronal differentiation of mouse bone marrow-derived mesenchymal stem cells (BM-MSCs).

**Materials and Methods:**

This experimental study was established in Kharazmi Univer- sity. BM was isolated from the bones of femur and tibia of 4-6-week old Naval Medical Research Institute (NMRI) mice, and the cells were cultured. The cells were divided into following 4 groups based on the applied treatments: I. control (no treatment), II. steroid hormones (β-estradiol, progesterone and testosterone), III. bFGF and IV. combination of steroid hormones and bFGF. Immunocytochemistry and flow cytometery analyses were applied for beta III-tubulin (β-III tubulin) and microtubule-associated proteins-2 (MAP-2) in 4 days of treatment for all groups.

**Results:**

The cells treated with combination of bFGF and steroid hormones represented more expressions of neural markers as compared to control and to other two groups treated with either bFGF or steroid hormones.

**Conclusion:**

This study showed that BM-MSCs can express specific neural markers after receiving bFGF pretreatment that was followed by sex steroid hormones treatment. More investigations are necessary to specify whether steroid hormones and bFGF can be considered for treatment of CNS diseases and disorders.

## Introduction

Bone marrow-derived mesenchymal stem cells ( BM-MSCs ) are multipotent adult stem cells with mesodermal origin. MSCs can be isolated from bone marrow, being expanded and genetically modified in culture medium in order to be prepared as cellular carriers for local or systemic treatments. BM-MSCs are considered as a potent cell source for this purpose, since they have a high potency in self-renewal, multi-differentiation, and producing functional structures *in vivo*. Studies have shown that BM cells have potential to differentiate into myocytes, hepatocytes and glial cells. Researchers reported that BM-MSCs of humans and mice can be induced and differentiate into neural cells under culture conditions (1,2). 

MSCs produce both growth factors and important cytokines which can facilitate the regeneration of damaged tissues. Growth factors are mitogenic polypeptides which play important roles in proliferation and differentiation of neuronal and astroglial cells in culture medium (3). 

Steroid hormones actively take part in bone metabolism, and have multiple regulatory functions indifferent cells, such as BM-MSCs. Dexamethasone ( DEX ) and estradiol are neurosteroids which play regulatory roles in neural cell lines, specifically in astroglial types (4). For instance, glucocorticoid ( GC ) has a key role in multi-differentiation of MSCs and can increase the potential of osteogenic, chondrogenic and adipogenic differentiation. These results suggest that steroid hormones can be considered as effective stimulators to improve the MSCs capacities and being useful in tissue engineering. DEX and estradiol interact and regulate cell function via estrogen and glucocorticoid receptors (5,6). These receptors are distributed differently based on gender in cardiac, brain and fat tissues (7). 

Estrogens interfere in neuronal and astroglial differentiation and are key hormones in neurodegenerative events (8). Estrogen shows regulatory functions via estrogenic receptors ( ER-a,-b ) available on some progenitor cells such as embryonic and MSCs. Estrogen can also promote the neural differentiation of embryonic stem cells (9). 

It was previously believed that the differentiation potential in stem cells is confined to the lineage specific and their tissue origin, whereas it has been reported that BM-derived cells do not show such limitation. These cells can be differentiated into neural cells, including neurons of different areas, when transplanted into human and mouse *in vivo* (10). It is important to be noticed that such a differentiation is dependent on experimental conditions and some factors remaining unknown. Recent findings have shown that the advantages, limitations and mechanisms of action of MSCs to treat some neurodegenerative disorders including Parkinson’s, Huntington’s and Alzheimer’s diseases, amyotrophic lateral sclerosis ( ALS ), multiple sclerosis ( MS ) and spinal cord lesions. Pluripotent and multipotent stem cells with differentiation capacities for neural phenotypes have been noticed through past decades. Recent studies have indicated the capability of BM cells in migrating towards the brain and gaining neuronal and glial characteristics. BM-MSCs can be induced by chemical compounds to express the markers of this lineage *in vitro* (11,12). Since, neurodegenerative diseases are progressively increasing these days, and proliferation and regeneration in central nervous system ( CNS ) is rare, cell therapy has become an attractive approach in neuroscience and there are so many attempts using different stem cells and biochemical factors to promote and induce neural differentiation (13). 

Various chemical inductors, such as butylhydroxytoluene, butylhydroxyanisol ( BHA ), dimethyl sulfoxide ( DMSO ), 2-mercaptoethanol, 3-isobutyl-1-methylxanthine ( IBMX ) and 5-azacytidine have been used alone or in combination. In some of these studies, differentiation was initiated by a 1-day first step using basic fibroblast growth factor ( bFGF ) as a pre-inductive molecule that has been previously described to control neural stem cell ( NSC ) self-renewal and differentiation (14,15). 

According to previous works, using bFGF as a pre-treatment agent seems to be proper for BMMSCs cultures. By adding this growth factor into culture medium, neurite-like extensions are recognizable after 4 days and fully developed after 7 days. However, from day 7, cells readopt a fibroblastoid shape, demonstrating the fact that these conditions are not able to make long-term differentiation, but the idea of using some growth factors as pre-treatments for researchers seems to be reasonable (14,15). Therefore, the present study aimed to investigate the effects of sex steroid hormones and bFGF on neuronal differentiation of mouse BM-MSCs. 

## Materials and Methods

This experimental study was established in research center of Kharazmi University and all the procedures were approved by the local Ethics Committee at Kharazmi University. Four-six-week old Naval Medical Research Institute ( NMRI ) mice ( purchased from Razi Institute ) were sacrificed by cervical dislocation. In sterile conditions, BM-MSCs were harvested from the bone marrow of the femurs and tibias, and the cells were plated into a T-25 flask ( SPL, Korea ) with dulbecco's modified Eagle's medium ( DMEM; GIBCO, USA ) containing 15% fetal bovine serum ( FBS; GIBCO, USA ) and 100 U/ml penicillin-streptomycin ( GIBCO, USA ) and incubated at 37˚C in a humidified atmosphere containing 5% CO_2_( Memmert, Germany ). After 48 hours, non-adherent cells were discarded and the medium was replaced every 3-4 days. 

The cells were characterized by flow-cytometric analysis of specific surface antigens of cluster of differentiation ( CD ) CD45, CD31, CD44, and CD29. When reaching 80% confluency, the cells were passaged, and after 2 passages, they were seeded in poly-lysine coated 24-well plates ( SPL, Korea ). The cells were incubated without any treatment and after 24 hours, they were divided into 4 groups and the treatments were administered for 4 days. The first group was considered as control group, with no treatment. The cells in second group received the combination of hormones, β-estradiol, progesterone and testosterone ( all purchased from Sigma, USA ), in equal concentrations of 90 µM/ each and the cells of group 3 were treated with bFGF ( 25 ng/ml; Sigma, USA ). The cells in group 4 received bFGF ( 25 ng/ml ) pretreatment for first two-day and hormones ( β-estradiol, progesterone and testosterone in equal concentrations of 90 µM/ each ) treatment for second two-day. Each group was treated for 4 days. Immunocytochemical and flow cytometric analysis were performed for neural markers of microtubule-associated proteins-2 ( MAP-2 ) and beta III-tubulin ( β-III-tubulin ), for all 4 groups after 4 days of treatment. 

## Results

Our findings showed that MSCs derived from bone marrow were in a heterogenous suspension phase with other cells in culture dish and gradually adhered to the flask surface. After 48 hours, the medium was replaced to remove the other undesired suspending cells. MSCs tend to form colonies and after some days, they reached their normal spindle shape in colonies. 

**Fig.1:**
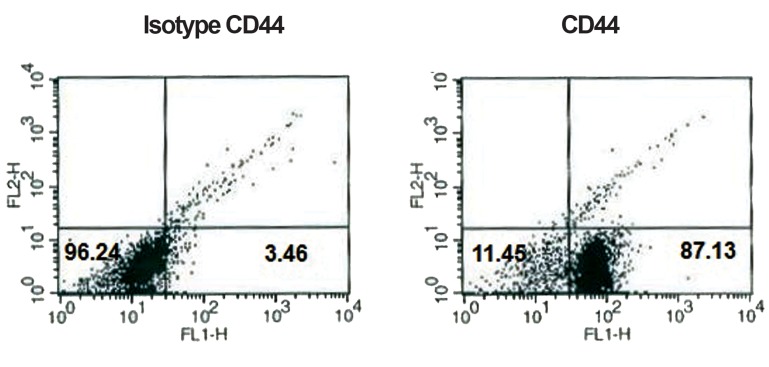
Flow cytometric results of BM-MSCs for surface antigen of CD44: 87.13% of cells expressed CD44. BM-MSCs; Bone marrow-derived mesenchymal stem cells and CD; Cluster of differentiation.

**Fig.2:**
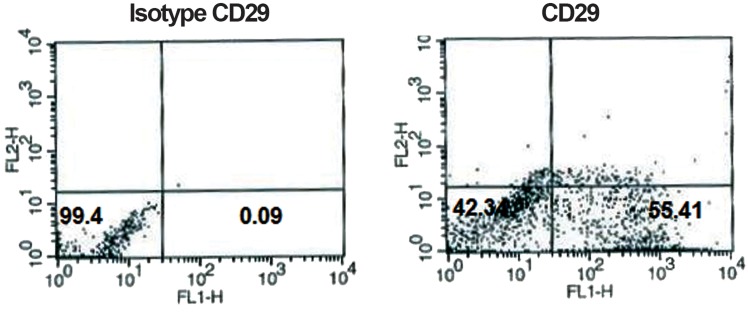
Flow-cytometric results of BM-MSCs for surface antigen of CD29: 55.41% of cells expressed CD29. BM-MSCs; Bone marrow-derived mesenchymal stem cells and CD; Cluster of differentiation.

**Fig.3:**
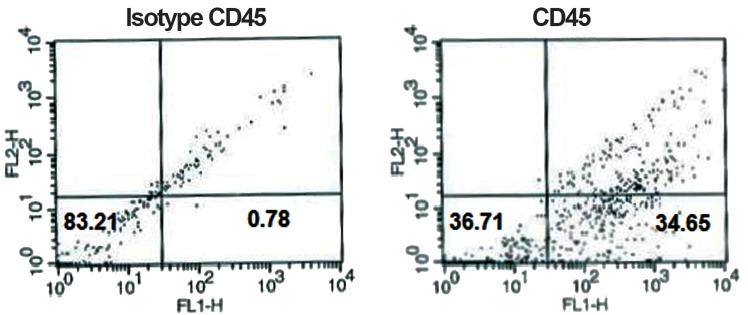
Flow-cytometric results of BM-MSCs for surface antigen of CD45: 34.65% of cells expressed CD45. BM-MSCs; Bone marrow-derived mesenchymal stem cells and CD; Cluster of differentiation.

**Fig.4:**
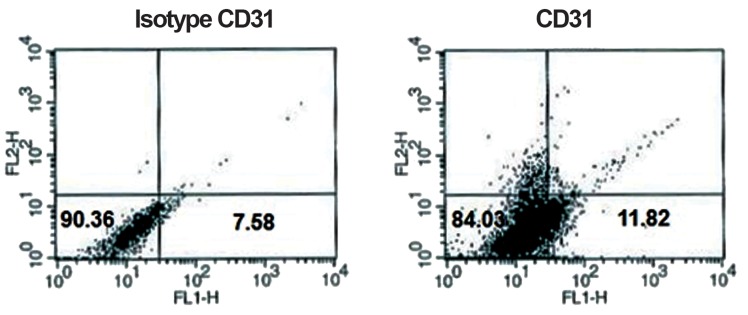
Flow-cytometric results of BM-MSCs for surface antigen of CD31: 11.82% of cells expressed CD31. BM-MSCs; Bone marrow-derived mesenchymal stem cells and CD; Cluster of differentiation.

Flow cytometry analysis of surface antigens revealed that cells were negative for CD45 and CD31, while positive for CD44 and CD29 (Figs.1-4). After 2 passages, the cells were transferred to 24-well plates and incubated for 24 hours with no treatment. Then the defined treatments were performed for 4 groups of cells. After 4 days, the cells were morphologically monitored and immunocytochemistry and flow cytometry were applied for specific neural markers of MAP-2 and β-IIItubulin. 

**Fig.5:**
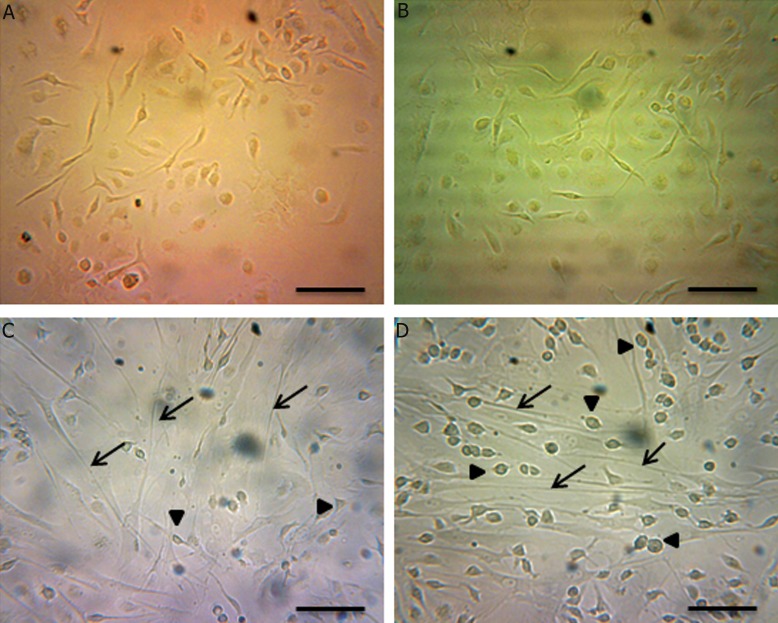
Mesenchymal stem cell cultures. Representative images from 4 experimental groups. A. Control group; was untreated and no special change is seen, B. Hormones group; treated with hormones, after 4 days no very obvious extensions are detectable, C. bFGF group; 4 days after bFGF treatment, extending processes are more clear and D. bFGF with hormones group; cells progressively developed neurite-like extensions. Some cells represented a long extension ( arrows ) from the small-cell body ( arrowheads ), scale bars=50 μm. bFGF; Basic fibroblast growth factor and Hormones; β-estradiol, progesterone and testosterone.

**Fig.6:**
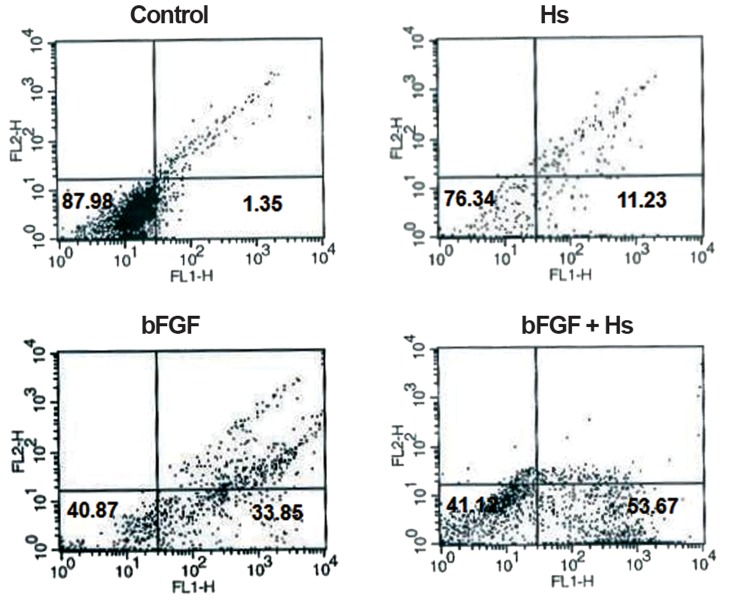
Flow-cytometric results of 4 experimental groups for β-III tubulin: the expression level was 1.35% in control group, 11.23% in hormones group, 33.85% in bFGF group and 53.67% in bFGF with hormones. bFGF; Basic fibroblast growth factor and Hs ( hormones ); β-estradiol, progesterone and testosterone.

**Fig.7:**
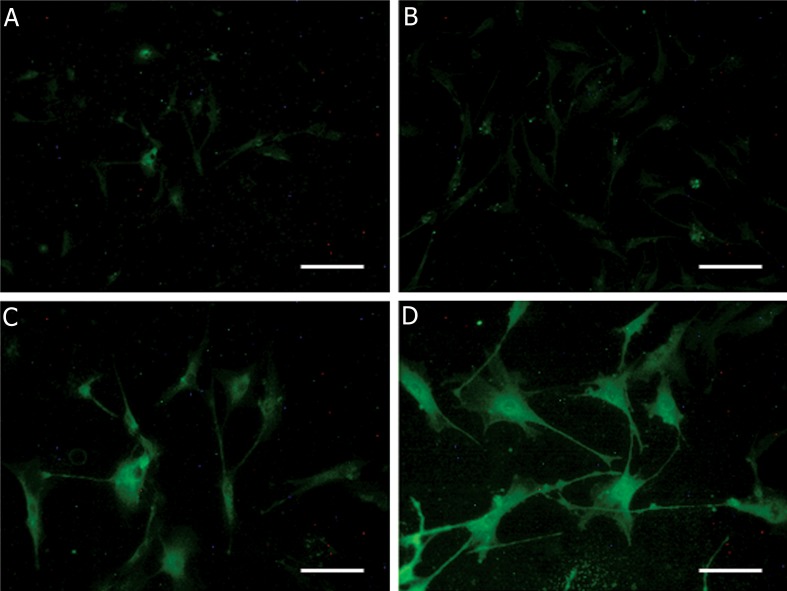
Fluorescent micrographs of cells in 4 experimental groups for cytosolic neural marker, β-III tubulin, conjugated with FITC. A. Control group; no special green stain can be seen ( scale bar=50 μm ), B. Hormones group; the cells are not stained well ( scale bar=50 μm ), C. bFGF group; the stain is well-detectable in cells, more than 2 previous groups ( scale bar=25 μm )and D. bFGF with hormones group; the cells are whole stained. The most staining level is seen, as compared with other groups ( scale bar=25 μm ). FITC; Fluorescein isothiocyanate, bFGF; Basic fibroblast growth factor and Hormones; β-estradiol, progesterone and testosterone.

**Fig.8:**
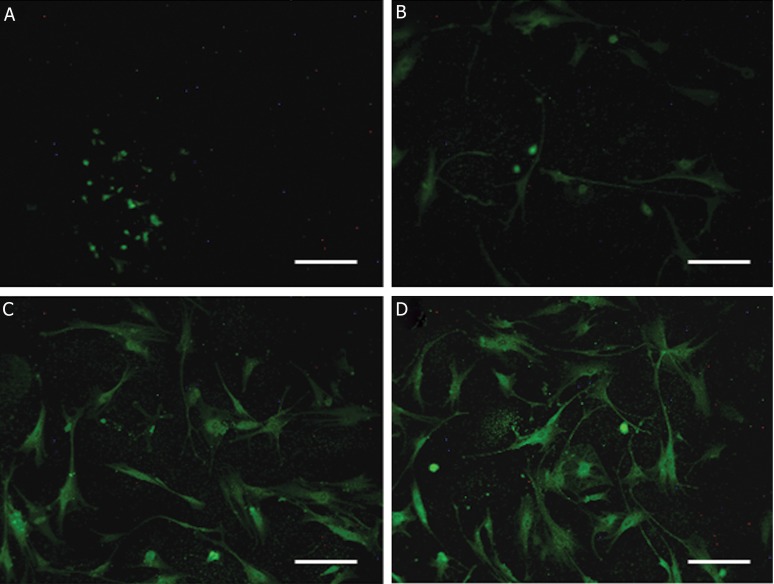
Fluorescent micrographs of cells in 4 experimental groups for cytosolic neural marker, MAP-2, conjugated with FITC. A. Control group; no special green stain can be seen, B. Hormones group; the cells are not stained well, C. bFGF group; the stain is well-detectable in cells, more than 2 previous groups and D. bFGF with hormones group; the cells are whole stained. The most staining level is seen, comparing with other groups ( scale bars=50 μm ). MAP-2; Microtubule-associated protein 2, FITC; Fluorescein isothiocyanate, bFGF; Basic fibroblast growth factor and Hormones; β-estradiol, progesterone and testosterone.

In control group with no treat, after 4 days, no special change was observed. Whereas, in group 4 with bFGF pretreatment, in first 2-day and hormones treatment, in second 2-day, neurite-like extensions were recognizable after 4 days. These changes in cell appearance were less and slighter seen in group 3 with bFGF and group 2 with hormones, respectively (Fig.5). In addition, molecular analysis of both neural markers, affirming morphological observations, revealed that the most expression level was in group 4 ( combination of bFGF and hormones ) and these markers showed less expression level in group 3 and group 2, respectively (Figs.6-8). 

## Discussion

BM-MSCs are proper options among different cell sources for tissue engineering and regenerative therapies. However, MSCs isolated from various origins show diverse potentials in differentiation. Little is known on the molecular mechanisms controlling MSCs differentiation, whilst these cells seem to have a great differentiation potential. Recent studies have found the ability of MSCs in differentiating into ectodermal ( neurons ) and endodermal ( hepatocytes ) tissues *in vitro* (16,17). 

Estrogen can effectively enhance the multidifferentiative potentials of human BM-MSCs *in vitro*, and specifically play key roles in tissue engineering and MSCs modification as different regulators (17). Studies have shown that 17-β-estradiol ( E2 ) effectively stimulate the proliferative potential of MSCs in human and mouse (18,19). Additionally, E2 have a high ability in supporting the differentiation potential of MSCs (20). Estrogens, affecting the asA B C D 34 troglial group (21), can permissively facilitate neurotrophic activities by genomic cross-talk with neurotrophin or growth factor regulative pathways (22). 

Some researchers have revealed that intravenously injected BM-MSCs migrate to the stroke injury site, differentiate into cells expressing neuronal markers and restore the damaged neural cells in rodents. Recent studies have indicated that female estrogenic hormones such as 17-β-estradiol affect the developing and adult brain function (23,24). Brannvall et al. (25) showed that E2 treatment for NSCs, cultured from rat brain in embryonic day 20, increased the proliferation and neural differentiation in these cells. For many years, it was supposed that sex steroids like estrogen, are synthesized in peripheral tissues including prostate, ovary, adrenal cortex, etc. and reach the brain through blood circulation and control sex performance or stress responses. Recently, it has been cleared that estrogen is also synthesized in hippocampal neurons of rat brain, and represents a broad spectrum of functions including neuroprotective effects and a stimulatory effect on cognitive function (26). 

However, there are some ambiguities regarding where estrogen synthesis site is in early developmental stages and it is still a question whether NSCs can synthesize the estrogens and start an autocrine mechanism themselves (27). 

Using a combination of sex steroids suggest a new approach in systemic regenerative therapies, neurodegenerative diseases and CNS disorders. In 2008, a research team using a specific combination of sex steroids observed neural differentiation in human ovarian epithelial stem cells. Their results showed that testosterone, progesterone and estradiol alone had no neural differentiative effect on these cells, whereas a combination of testosterone and progesterone, after a pre-treatment with estradiol, differentiated many human ovarian epithelial stem cells and porcine granulosa cells into NSCs and neuronal cells, within one to three hours (28). These observations indicated that systemic treatment with sex steroids and their mixture might be likely effective in the treatment or prevention of degenerative CNS disorders. The ovarian stem cell cultures that are readily obtainable from human ovaries, regardless of the women’s age, can be considered as a good potential to produce NSCs for autologous regenerative treatment of neural diseases in aging women. They reported that a proper mixture of sex steroids can likely be used to differentiate the adult bone marrow stem cells or peripheral blood cells into autologous NSCs and stimulate their neuronal differentiation after homing in CNS (29). 

In addition, the studies have shown that when mouse MSCs transport into culture medium ( *in vitro* ) and remain for a long time, they might lose many features of stemness and gain some novel characteristics to promote differentiation. Some researchers in 2004 observed that MSCs, after many passages *in vitro*, without any specific treatment expressed neural lineage markers. Although such changes in molecular marker expression are usually transient and is not considered as stable differentiation, they might mislead the researchers. These cells after being isolated from their main niche, full of normal signals and specific conditions, should not be maintained in culture medium for a long time, and the treatment should be administered soon after reaching a homogenous cell source (30,31). 

## Conclusion

Our data suggested that sex steroid hormones supplement enhances the neural differentiation effects of bFGF on cultured BM-MSCs. These findings could be used in the cultivation of BMMSCs for cell-based strategies for CNS disease treatment. This study showed that BM-MSCs can express specific neural markers after receiving bFGF as a pretreatment that is followed by receiving sex steroid hormones as treatment. It can be concluded that some steroid hormones may be considered as a drug for treatment of CNS diseases and disorders in future studies. 

## References

[1] Arthur A, Zannettino S, Gronthos S (2009). The therapeutic applications of multipotential mesenchymal/stromal stem cells in skeletal tissue repair. J Cell Physiol.

[2] Sanchez-Ramos J, Song S, Cardozo-Pelaez F, Hazzi C, Stedeford T, Willing A (2000). Adult bone marrow stromal cells differentiate into neural cells in vitro. Exp Neurol.

[3] Bronzi D, Bramanti V, Tomassoni D, Laureanti F, Grasso S, Li Volsi G (2010). Neural markers expression in rat bone marrow mesenchymal stem cell cultures treated with neurosteroids. Neurochem Res.

[4] Avola R, Di Tullio MA, Fisichella A, Tayebati SK (2004). Glial fibrillary acidic protein and vimentin expression is regulated by glucocorticoids and neurotrophic factors in primary rat astroglial cultures. Clin Exp Hypertens.

[5] Cuzzocrea S, Bruscoli S, Crisafulli C, Mazzon E, Agostini M, Muia C (2007). Estrogen receptor antagonist fulvestrant (ICI 182, 780) inhibits the anti-inflammatory effect of glucocorticoids. Mol Pharmacol.

[6] Mattsson C, Olsson T (2007). Estrogens and glucocorticoid hormones in adipose tissue metabolism. Curr Med Chem.

[7] Fadini GP, De Kreutzenberg S, Albiero M, Coracina A, Pagnin E, Baesso I (2008). Gender differences in endothelial progenitor cells and cardiovascular risk profile: the role of female estrogens. Arterioscler Thromb Vasc Biol.

[8] Bramanti V, Bronzi D, Raciti G, Avitabile M, Avola R (2007). Neurosteroid-growth factor cross-talk induces up and down regulation of GFAP and vimentin expression in serum free astrocyte cultures. Ital J Biochem.

[9] Ramalho AC, Jullienne A, Couttet P, Graulet AM, Morieux C, de Vernejoul MC (2001). Effect of oestradiol on cytokine production in immortalized human marrow stromal cell lines. Cytokine.

[10] Cogle CR, Yachnis AT, Laywell ED, Zander DS, Wingard JR, Steindler DA (2004). Bone marrow transdifferentiation in brain after transplantation: a retrospective study. Lancet.

[11] Brazelton TR, Rossi FM, Keshet GI, Blau HM (2000). From marrow to brain: expression of neuronal phenotypes in adult mice. Science.

[12] Torrente Y, Polli E (2008). Mesenchymal stem cell transplantation for neurodegenerative diseases. Cell Transplant.

[13] Sadan O, Melamed E, Offen D (2009). Bone-marrow-derived mesenchymal stem cell therapy for neurodegeneratine diseases. Expert Opin Biol Ther.

[14] Rismanchi N, Floyd CL, Berman RF, Lyeth BG (2003). Cell death and long-term maintenance of neuron-like state after differentiation of rat bone marrow stromal cells: a comparison of protocols. Brain Res.

[15] Tropel P, Platet N, Platel JC, Noel D, Albrieux M, Benabid AL (2006). Functional neuronal differentiation of bone marrow-derived mesenchymal stem cells. Stem Cells.

[16] Hong L, Colpan A, Peptan IA (2006). Modulations of 17-beta estradiol on osteogenic and adipogenic differentiations of human mesenchymal stem cells. Tissue Eng.

[17] Brooke G, Cook M, Blair C, Han R, Heazlewood C, Jones B (2007). Therapeutic applications of mesenchymal stromal cells. Semin Cell Dev Biol.

[18] Di Silvio L, Jameson J, Gamie Z, Giannoudis PV, Tsiridis E (2006). In vitro evaluation of the direct effect of estradiol on human osteoblasts (HOB) and human mesenchymal stem cells (h-MSCs). Injury.

[19] Zhou S, Greenberger JS, Epperly MW, Goff JP, Adler C, Leboff MS (2008). Age-related intrinsic changes in human bone-marrow-derived mesenchymal stem cells and their differentiation to osteoblasts. Aging Cell.

[20] Hong L, Sultana H, Paulius K, Zhang G (2009). Steroid regulation of proliferation and osteogenic differentiation of bone marrow stromal cells: a gender difference. J Steroid Biochem Mol Biol.

[21] JungTestas I, Renoir M, Bugnard H, Greene GL, Baulieu EE (1992). Demonstration of steroid hormone receptors and steroid action in primary cultures of rat glial cells. J Steroid Biochem Mol Biol.

[22] Smith CL (1998). Cross-talk between peptide growth factor and estrogen receptor signaling pathways. Biol Reprod.

[23] Jiang Y, Jahagirdar BN, Reinhardt RL, Schwartz RE, Keene CD, Ortiz-Gonzalez XR (2002). Pluripotency of mesenchymal stem cells derived from adult marrow. Nature.

[24] McEven B (2002). Estrogen actions throughout the brain. Recent Prog Horm Res.

[25] Brannvall K, Korhonen L, Lindholm D (2002). Estrogen-receptor-dependent regulation of neural stem cell proliferation and differentiation. Mol Cell Neurosci.

[26] Tsurugizawa T, Mukai H, Tanabe N, Murakami G, Hojo Y, Kominami S (2005). Estrogen induces rapid decrease in dendritic thorns of CA3 pyramidal neurons in adult male rat hippocampus. Biochem Biophys Res Commun.

[27] Kim K, Son TG, Kim SJ, Kim HS, Kim TS, Han SY (2007). Suppressive effects of bisphenol A on the proliferation of neural progenitor cells. J Toxicol Environ Health A.

[28] Okada M, Murase K, Makino A, Nakajima M, Kaku T, Furukawa S (2008). Effects of estrogens on proliferation and differentiation of neural stem/progenitor cells. Biomed Res.

[29] Bukovsky A, Caudle MR, Svetlikova M (2008). Steroid-mediated differentiation of neural/neuronal cells from epithelial ovarian precursors in vitro. Cell Cycle.

[30] Ahmadbeigi N, Soleimani M, Gheisari Y, Vasei M, Amanpour S, Bagherizadeh I (2011). Dormant phase and multinuclear cells: two key phenomena in early culture of murine bone marrow mesenchymal stem cells. Stem Cells Dev.

[31] Tondreau T, Lagneaux L, Dejenefle M, Massy M, Mortier C, Delforge A (2004). Bone marrow-derived mesenchymal stem cells already express specific neural proteins before any differentiation. Differentiation.

